# Approach to hematopoietic cell transplant candidates with respiratory viral detection

**DOI:** 10.3389/fped.2023.1339239

**Published:** 2024-01-18

**Authors:** Sara R. Kim, Alpana Waghmare, Diego R. Hijano

**Affiliations:** ^1^ Division of Pediatric Infectious Diseases, Seattle Children’s Hospital, Seattle, WA, United States; ^2^ Department of Pediatrics, University of Washington, Seattle, WA, United States; ^3^ Vaccine and Infectious Diseases Division, Fred Hutchinson Cancer Center, Seattle, WA, United States; ^4^ Departments of Infectious Diseases, St. Jude Children’s Research Hospital, Memphis, TN, United States; ^5^ Department of Pediatrics, University of Tennessee Health Sciences Center, Memphis, TN, United States

**Keywords:** respiratory virus, children, immunocompromised, cancer, hematopoietic cell transplant

## Abstract

The management of respiratory viruses prior to hematopoietic cell transplant (HCT) can be controversial and requires special consideration of host factors, transplant parameters, and the specific respiratory virus (RV). In the setting of adenovirus (ADV), human metapneumovirus (HMPV), influenza, parainfluenza virus (PIV), and respiratory syncytial virus (RSV) detection prior to hematopoietic cell transplant (HCT), clinical practice guidelines recommend transplant delay when possible; however, there is much more ambiguity when other respiratory viruses, such as seasonal coronaviruses (CoVs), human rhinovirus (HRV), and SARS-CoV-2, are detected. Our aims for this review include detailing clinical practical guidelines and reviewing current literature on pre-transplant respiratory viral infections (RVIs), including antiviral therapies and prevention strategies, when available. We will center our discussion on three representative clinical scenarios, with the goal of providing practical guidance to clinicians.

## Introduction

Patients who will receive or have undergone hematopoietic cell transplant (HCT) are at high risk of experiencing severe illness due to respiratory viral infections (RVIs) caused by chemotherapy-induced myelosuppression, impaired T-cell immunity, lymphopenia, and the use of immunosuppressive regimens (1–6). Prompt and accurate identification of viral respiratory pathogens is crucial for ensuring the best possible care for these patients. The symptoms of different RVIs are similar and do not help to differentiate pathogens. Therefore, patients suspected of having a RVI should be tested for a range of pathogens, allowing for targeted therapy where treatments exist, prompt implementation of appropriate infection control measures, appropriate monitoring of secondary infections, and minimization of empirical treatment for alternative conditions of concern.

## Case 1: Should asymptomatic patients be tested for RVIs prior to HCT?

One-year-old child with acute megakaryoblastic leukemia is a candidate for unrelated, mismatched cord transplant. Seven days before the start of conditioning, the patient tested positive for human rhinovirus (HRV) in routine multiplex PCR testing. The patient was asymptomatic at the time of positive PCR results and transplant conditioning.

## Diagnostic considerations

### Routine vs. symptom driven testing

The role of systematic testing of patients before transplant in the absence of symptoms has been proposed and implemented by many groups (7–9). This approach provides an opportunity for treatment if available, as well as implementation of infection prevention measures to mitigate spread within the unit. With the availability of highly sensitive multiplexed PCR assays, this practice has led to many questions, particularly related to the clinical relevance of multiple viral co-infections and whether changes in management (delay, treatment when available) should be recommended for asymptomatic detection of respiratory viruses (10–13). This is particularly challenging in immunocompromised children, in whom respiratory viruses are detected more frequently than in adults, and in those who can shed viruses for prolonged periods of time (14–16). In addition, transplant delay decisions are affected by the type of conditioning, transplant, donor, specific virus, and availability of antiviral agents. It is well established that myeloablative conditioning, allogeneic transplant, and matching unrelated or haploidentical donors are associated with an increased risk of poor outcome (17–25). A classification of respiratory viruses based on their potential for severe diseases and complications in these populations has been proposed. Respiratory syncytial virus (RSV), human metapneumovirus (HMPV), parainfluenza virus (PIV), influenza A and B, and adenovirus (ADV) have been grouped among the viruses most likely to cause severe disease, whereas human rhinoviruses (HRVs), human seasonal coronaviruses (CoVs), and human bocavirus are less frequently associated with severe disease (7, 26, 27). In a study of pediatric HCT recipients, pre-HCT detection of respiratory viruses, including HRVs, was associated with increased hospitalization within the first 100 days (7). Campbell et al. performed a large prospective study of children and adults undergoing HCT. They found that asymptomatic patients who had surveillance samples viral testing performed and detected, had no increase in bronchoscopy incidence or overall mortality, whereas those patients with respiratory symptoms had increased mortality compared to symptomatic individuals with no virus detective, irrespective of the virus (26). These data suggest that respiratory viruses, particularly in the presence of symptoms, may affect the transplant outcomes. Although there is a clear benefit from testing every patient with symptoms, the use of asymptomatic viral surveillance testing prior to HCT remains controversial and institutions vary in their approach.

### Molecular methods: multiplex vs. targeted assays

Molecular amplification techniques have become the gold standard for detecting and identifying viruses, surpassing cell culture as the preferred method (28, 29). Antigen-based detection is commonly used in acute care settings because of its faster turnaround time, but it has lower sensitivity and specificity than molecular methods. Polymerase Chain Reaction (PCR) remains the most widely used amplification technique, and several Food and Drug Administration (FDA) approved tests are available to detect the most common respiratory viral pathogens. These tests range from single-analyte assays to multiplex panels (30–34). Multiplex PCR enables the amplification of multiple target sequences in a single reaction. Multiplex assays are advantageous because they save time, cost, and samples. However, multiplexing has limitations, particularly in terms of design, optimization, and the need for modifications. Targeted PCR assays are less complex, and there is no risk of competition between targets during PCR. However, multiple reactions may be required to detect multiple targets, which can result in higher costs for materials and labor, as well as the consumption of more samples per reaction (28–31, 33–36). A multiplex broad panel can be beneficial for immunocompromised individuals because symptoms can be unusual or subtle, and most of these viruses will present in a similar way, making it difficult to clinically diagnose the cause of the illness. Additionally, co-infections with multiple viruses are possible, particularly in children (8, 36, 37). The benefits of multiplex PCR testing outweigh the latter point and are recommended when available.

### Quantitative methods

Viral load determination in the blood and the clinical management of viruses, such as cytomegalovirus or Epstein-Barr virus, has been facilitated by the implementation of validated quantitative assays. Furthermore, the use of WHO standards has made reporting and comparison across assays possible, facilitating clinical studies and advancing the field (38–40). Currently, no quantitative respiratory viral assays are available for clinical care. With the emergence of SARS-CoV-2, many groups have started using cycle threshold (Ct) values as a surrogate for viral RNA quantity in clinical samples to guide clinical decisions (41–45). The use of RT-PCR Ct values as a substitute for RNA concentration may result in inaccuracies due to a non-linear correlation with the quantity of viral RNA load within the analytical measurement range (AMR) of a given assay (46–48). Within-assay variability across the AMR paired with poor inter-assay agreement and lack of linear correlation limits the generalizability of the results (47). While numerous scientific societies, including the Infectious Diseases Society of America (IDSA) and the Association of Molecular Pathology (AMP), have advised caution when presenting clinical data that utilize Ct values or rely on Ct values to determine active infection, disease severity, or transmissibility, the utilization of Ct values in these contexts persists (49, 50). Progress in quantifying viral loads in respiratory samples will advance if a model similar to that of double-stranded DNA viruses is developed.

### Shedding

The development of molecular assays has led to an increase in the duration of detectable viral shedding, which can be prolonged in immunocompromised patients [16–18]. Decreased antiviral immunity due to underlying disease conditions and treatments such as high-dose steroids, myeloablative conditioning, and post-engraftment immunosuppression increases the risk of prolonged viral shedding (14–16). In addition, the majority of patients who shed respiratory viruses for over 4 weeks are allogeneic transplant recipients, and those with lower respiratory tract infection (LRTI) tend to have even longer periods of asymptomatic shedding (51). Several groups have reported prolonged viral shedding in HCT recipients, with a median duration of 5 weeks for HRVs, 4 weeks for CoVs, and 2 weeks for PIV. Some patients experienced prolonged shedding for more than 3 months (3, 52–54). Immunocompromised individuals with prolonged shedding are reservoirs for viral mutations, often resulting in antiviral drug resistance (6, 55–62). Although the risk of nosocomial spread during periods of asymptomatic shedding is unknown, outbreaks of these viruses in transplant units have been described, and the general recommendation is to continue contact isolation and droplet throughout the duration of the illness or hospitalization, and per institutional guidelines thereafter (1, 63–72). Finally, the clinical significance of prolonged viral shedding before transplant is unclear and it is unknown whether it increases the risk of recurrent symptoms, severe disease, or death. Therefore, decisions around delaying or proceeding with HCT in asymptomatic shedding require further study.

### Summary: There is insufficient evidence to recommend for or against viral testing prior to HCT in asymptomatic patients. We recommend multiplex viral PCR testing in any patient with respiratory symptoms prior to HCT

## Case 2: Strategies to help inform proceeding or delay HCT

A 6-year-old child with polycythemia vera was evaluated for HCT from an unrelated, matched donor. Two weeks before the planned start of conditioning therapy, the patient developed cough and rhinorrhea. A nasal swab was collected for respiratory viral testing, and the results were positive for the parainfluenza virus 3.

### Delay considerations

HCT candidates that develop symptomatic RVIs prior to transplant represent a challenging clinical scenario. There is concern for poor transplant outcomes related to the infection itself, and a delay in transplant may be considered until the patient has recovered. However, the primary diagnosis for which transplant is being performed may include aggressive hematologic malignancies, for which a delay represents a significant risk of disease progression. Furthermore, depending on the cell source, there may be logistical considerations regarding donor and/or product availability, as well as other scheduling considerations. Given this complexity, transplant delay decisions must be approached carefully, considering both infection and disease status, along with other patient-specific factors.

Given that current guidelines recommend considering transplant delay when RVs are detected in HCT candidates (12, 73, 74), most transplant centers will consider transplant delay for viruses associated with severe diseases, such as RSV, influenza, HMPV, ADV, PIV, and SARS-CoV-2. This is an important consideration when interpreting contemporary studies to evaluate the impact of pre-HCT RVIs on transplant outcomes, as most are conducted in the setting of a recommendation to delay HCT. Campbell et al. collected pre-HCT and weekly post-HCT nasal washes through day 100 from 458 adults and children undergoing HCT with and without symptoms. Samples were tested by multiplex PCR. Patients with a RV detected prior to transplant had fewer days alive and out of the hospital, and higher mortality at day 100 after transplant compared to patients without a virus detected (26). Importantly, this effect was observed in patients with HRV detected alone, the most commonly detected RV. A follow up study in a larger cohort of 1,643 adult HCT recipients who underwent testing with multiplex PCR if they had symptoms, sought to further characterize the risk by stratifying by the presence of symptoms and the location of the infection (upper vs. lower respiratory tract infection) (9). Here, pre-HCT upper respiratory tract infection with any RV, including HRV, was not associated with increased overall mortality or fewer days alive and out the hospital. However, LRTI due to any virus, including HRV alone, were associated with increased overall mortality and fewer days alive and out of the hospital. The effect was driven primarily by allogeneic HCT recipients receiving myeloablative conditioning.

Data on pediatric HCT recipients are limited. As in adults, HRV is the most common RV detected, both before and after transplant. In a study of pediatric HCT candidates who underwent multiplex PCR surveillance testing pre-HCT, regardless of symptoms, those with pre-HCT HRV detection (*N* = 22) had outcomes at day 100 post-HCT comparable to those that were negative for all viruses (*N* = 34), including requirement for ventilatory support, length of stay, mortality, or days alive and out of the hospital (75). Children with LRTI were found to have fewer days alive and out of the hospital within the first 100 days post-HCT. In another study of 535 children undergoing allogeneic HCT, of which 75 had a positive RV test prior to transplant, patients with a positive RV result had lower overall survival and increased transplant related mortality (13). HRV was the most commonly detected virus, although it represented only 29% of the cases. In comparing patients in which transplant was delayed vs. not, the delayed patients had improved survival, although details on the reasons for delay are not clear. Though these data are suggestive of an association between pre-HCT RV detection and poor post-HCT outcomes, larger studies are needed to further define the effect of specific viruses, presence of symptoms, infection location, and transplant factors including cell source and conditioning regimen.

Our practice is to consider delay of transplant in any HCT recipient who have evidence of LRTI due to any RV, and in patients with symptomatic upper respiratory tract infection due to RSV, influenza, HMPV, ADV, PIV or SARS-CoV-2. Patients infected with treatable viruses should be started on therapy as outlined below. Delays are not routinely recommended for patients with HRV or non-SARS-CoV-2 coronaviruses. Individual patient factors including severity of underlying disease, urgency of transplant, level of anticipated immunosuppression, and other logistical issues were taken into consideration, and multidisciplinary approach was taken. If delay is feasible, the recommendation is to postpone HCT by at least 2 weeks with resolution of, or at least significant improvement in, symptoms. Ideally, the RV should no longer be detectable; however, this is not always achievable (Table 1). If viral load data is available, a decrease in viral load may also be reassuring to proceed to transplant, although no data to support this are currently available. Similarly, data on the optimal duration of delay are not known.

**Table 1:** Recommendations for respiratory viral infections before transplant.

Virus	Recommendation for URTI	Recommendation for LRTI
RSV	Delay transplant if possible	Delay transplant; consider ribavirin^a^
	If not possible to delay, consider oral ribavirin^a^	
Influenza virus	Delay transplant if possible and treat^b^	Delay transplant and treat^b^
	If not possible to delay, treat^b^	
SARS-CoV-02	Delay transplant if possible and treat^b^	Delay transplant and treat^b^
	If not possible to delay, treat^b^	
Parainfluenza virus	Delay transplant if possible	Delay transplant
	If not possible to delay, supportive care	
Metapneumovirus	Delay transplant if possible	Delay transplant; no data on ribavirin
	If not possible to delay, supportive care	
Rhinovirus	Delay transplant if possible, especially if symptomatic	Delay transplant if possible
Coronavirus	Delay transplant if possible, especially if symptomatic	Delay transplant if possible
Bocavirus	No data	No data

^a^ Benefit and dosing of oral ribavirin in pediatric patients is not clearly understood.

^b^ See influenza and SARS-CoV-2 treatment section in Table 2.

### Summary: Available data recommend delaying HCT whenever feasible in the setting of a symptomatic LRTI for any RV and symptomatic URTI due to RSV, influenza, HMPV, ADV, PIV, or SARS-CoV-2

## Case 3: Preventative and therapeutic interventions for RVIs before HCT

14-year-old adolescent with relapsed ALL, who has a planned lymphodepletion in 2 weeks for chimeric antigen receptor (CAR) *T*-cell therapy, presents to clinic with a mild sore throat, cough, and low-grade fever. Physical examination revealed mild posterior pharyngeal erythema and rhinorrhea, but the lungs were clear to auscultation bilaterally with no oxygen requirement. A nasopharyngeal swab for a multiplex PCR of different respiratory viruses was positive for SARS-CoV-2. Prior to diagnosis, the patient was administered voriconazole for antifungal prophylaxis. While outpatient, the adolescent was started on ritonavir boosted nirmatrelvir for 5 days. After completing therapy, repeat SARS-CoV-2 PCR remained positive. Voriconazole was continued without any modifications, and hepatic function was monitored during treatment without any notable changes. Due to persistent SARS-CoV-2 positivity, the decision was made to delay HCT.

### Treatment considerations for a subset of viruses

There are limited therapies available to prevent and/or treat RVIs in HCT recipients (Table 2). Most studies have been conducted in adults, immunocompetent children, and/or during the post-HCT period; therefore, the efficacy of these therapies is inferred in pediatric HCT candidates. In this section, we review the prophylactic and therapeutic alternatives available for children undergoing HCT. Of note, there are no US-market-approved therapies available for HMPV, PIV, HRV, or non-SARS-CoV-2 COVs.

**Table 2:** Available therapeutic agents for respiratory viruses.

Antiviral	Route	Age/weight	Dosage	Duration	Side effects
Influenza^a^					
Oseltamivir	PO	0–8 months	3.0 mg/kg per dose, twice daily	5 days	Nausea, vomiting, headache, skin reactions, diarrhea
		9–11 months	3.5 mg/kg per dose, twice daily	5 days	
		≥12 months, ≤15 kg	30 mg, twice daily	5 days	
		≥12 months, >15 kg–23 kg	45 mg, twice daily	5 days	
		≥12 months, >23 kg–40 kg	60 mg, twice daily	5 days	
		≥12 months, >40 kg	75 mg, twice daily	5 days	
Zanamivir	Inhaled	≥7 years	10 mg (2.5-mg inhalations) twice daily	5 days	Bronchospasms, skin reactions
Peramivir	IV	6 months–12 years	12 mg/kg dose (600 mg maximum) over 15–30 min	5 days	
		≥13 years	600 mg dose over 15–30 min	5 days	
Baloxavir marboxil	PO	≥5 years, <20 kg	2 mg/kg per dose	1 day	Nausea, vomiting, diarrhea
		≥5 years, 20–<80 kg	40 mg dose	1 day	
		≥5 years, ≥80 kg	80 mg dose	1 day	
SARS-CoV-2^b^					
Remdesvir	IV	3 kg–<40 kg	5 mg/kg once on day 1 then 2.5 mg/kg once daily	3–10 days	Allergic reaction, transaminitis, nausea
		≥40 kg	200 mg once on day 1 then 100 mg once daily		
Paxlovid	PO	≥12 years, >40 kg	300 mg (nirmatrelvir)/100 mg (ritonavir) twice daily	5 days	Allergic reaction, liver toxicity, altered sense of taste, diarrhea
Respiratory syncytial virus					
Ribavirin	Inhaled	Infants and children	Continuous: 6 g administered over 12–18 h	3–7 days	Hemolytic anemia, bronchospasm, deterioration in pulmonary function, rash, conjunctival irritation, liver and renal toxicity
			Intermittent: 2 g over 2–3 h 3 times daily (113–115)	2–10 days	
	PO	Adults	20–30 mg/kg/day in 2–4 divided doses; round to nearest 200 mg dose (max dose: 1,800 mg/day)^c^	5–10 days	

^a^ Adapted from 2023 AAP Influenza Guidelines (76).

^b^ Adapted from NIH COVID Treatment Guidelines (77).

^c^ Off-label use and no established consensus for oral ribavirin dosing (78, 79).

#### Influenza

Cancer patients and HCT recipients are at high risk for influenza-related complications, including severe pneumonia and acute respiratory distress syndrome (80); therefore, annual vaccination and early initiation of antiviral therapy are recommended for maximal protection. Immunocompromised patients may present atypically and may shed influenza for a prolonged period. Exposure to influenza antivirals in the setting of prolonged shedding can promote antiviral resistance (6). With emerging resistance, the landscape of influenza antivirals continues to evolve, but the currently available influenza antivirals in the market are neuraminidase inhibitors (NAI) and one endonuclease inhibitor.

The first-line therapy for influenza are neuraminidase inhibitors (NAI), which are active against Influenza A and B (55, 81, 82). Oseltamivir (Tamiflu) is an oral NAI approved for children ≥2 weeks of age. It is generally well-tolerated, with nausea, emesis, and diarrhea being the most common side effects reported. Zanamivir, an inhaled NAI, is FDA approved for children ≥7 years but should be used in caution in patients with chronic lung disease and requires appropriate inhaler education. Parenteral NAIs include Zanamivir (currently in clinical trials) and Peramivir (approved as a single dose in children ≥6 months of age). In high-risk children, clinical guidelines recommend the treatment of influenza with NAIs, even if the symptom onset was greater than 48 h (76, 80). Treatment for uncomplicated influenza is recommended for 5 days but can be extended to 10 days in immunocompromised hosts (80).

Endonuclease inhibitors represent a new class of influenza antivirals that inhibit viral replication. Baloxavir marboxil (baloxavir) can be orally administered as a single dose in children ≥12 years of age and has been shown to be noninferior to NAIs in immunocompetent individuals with uncomplicated influenza (83, 84). However, the Centers for Disease Control and Prevention (CDC) does not recommend baloxavir in immunocompromised individuals due to limited studies and concerns regarding the development of resistance (85); baloxavir has been used in HCT recipients in the setting of NAI-resistant variants, severe infection, or failed first-line therapy (86–88). However, it should be avoided as stand-alone therapy. Combining baloxavir with NAIs, albeit well-tolerated, did not result in superior clinical outcomes compared with NAIs alone in patients hospitalized with severe influenza (89). Additional studies are needed to determine the role of combination therapy in immunocompromised children.

#### Respiratory syncytial virus

Despite the disease burden in both immunocompetent children and immunocompromised HCT recipients, there are no targeted antivirals against RSV currently available in the market. Ribavirin, a broad-spectrum guanosine nucleoside analogue, can inhibit viral replication of different RNA and DNA viruses, including RSV. Although inhaled ribavirin is FDA-approved for the treatment of RSV LRTI in pediatric patients, a systematic review of ribavirin use in infants with RSV LRTI did not lead to a decrease in mechanical ventilation or hospitalization in immunocompetent infants (90). However, a separate systematic review and meta-analysis of RSV LRTI, that included HCT recipients, showed a statistically significant decrease in mortality in mostly adult patients with hematologic disorders (91). Although there are a lack of randomized control trials prescribing ribavirin to immunocompromised and HCT recipients on ribavirin, the consensus and recommendations from international guidelines are to treat HCT recipients with RSV LRTI with inhaled or systemic ribavirin (12). However, the cost of aerosolized ribavirin ($29,953 per day) (92) and the mode of administration can be a barrier to treatment (78, 93, 94), and the IV ribavirin can only be obtained through an FDA Emergency Investigationa New Drug (EIND) application. Alternatively, oral ribavirin has been used in high-risk adult patients with RSV infection and has shown similar outcomes to aerosolized ribavirin in HCT recipients and immunocompromised patients (79, 93, 95–97). Considering its lower cost and ease of administration, oral ribavirin is a potential alternative for selected candidates. The safety profile often limits its use; hemolytic anemia, nephrotoxicity, drug rash, lactic acidosis, and altered mental status have been described with oral ribavirin. In addition, a boxed warning exists regarding the teratogenic effects of ribavirin observed in animal studies. Pregnancy should be avoided during and for 6 months after treatment in both female patients and the female partners of male patients treated with ribavirin (98–100).

#### SARS-CoV-2

Similar to influenza, the recommendation is to identify SARS-CoV-2 early in immunocompromised patients to provide prompt treatment before progression to COVID-19. Patients with hematologic malignancies are considered to be at a high risk for progression, and antiviral therapy should be considered in the HCT candidates. Ritonavir-boosted nirmatrelvir (Paxlovid) has an FDA EUA for treatment of non-hospitalized, high-risk adolescents greater than 12 years of age and weighing greater than or equal to 40 kg (77). In a randomized control trial of adults at risk for severe COVID-19, the EPIC-HR trial found an 89% relative risk reduction in COVID-19-related hospitalization or all-cause mortality in subjects receiving Paxlovid compared to placebo (101). A separate European study of patients with hematologic malignancies showed decreased mortality with Paxlovid use (102). However, Paxlovid has several drug-drug interactions due to ritonavir being a strong cytochrome P450 (CYP) 3A4 inhibitor; therefore, a thorough review of the patient's medication history is needed to assess potential changes, modifications, or discontinuation of certain drugs while receiving therapy (77). In reference to case 3, the patient was on voriconazole, which is also a CYP3A4 inhibitor, but based on the NIH guidelines, it is not a contraindication for Paxlovid administration (77). Given the oral formulation and the widespread availability of Paxlovid in local pharmacies, the patient was given Paxlovid for ease of administration and to avoid further hospital visits.

Remdesivir is a nucleotide prodrug of an adenosine analog that binds to viral RNA-dependent RNA polymerase and inhibits viral replication. It is approved by the FDA for treatment of COVID-19 in pediatric patients older than 28 days and weighing greater than or equal to 3 kg. In the PINETREE trial, patients at high risk for progression, including patients with hematologic malignancies, that received remdesivir had a decreased risk for hospitalization within 14 days after infection; however, there were no deaths reported in either the placebo or remdesivir group at 28 days post infection (103). As an alternative to Paxlovid, especially in patients with drug-drug interactions, remdesivir can be administered intravenously in the outpatient setting on 3 consecutive days; however, this may be logistically difficult in low-resource settings.

For high-risk children (i.e., immunocompromised) 12–18 years of age hospitalized for COVID-19, remdesivir should considered. If these patients require oxygen, remdesivir should be given for 5 days or until hospital discharge, whichever comes first. However, treatment course may be extended for up to 5 additional days (total duration of 10 days) (77). Dexamethasone is indicated when oxygen supplementation is needed or with increasing respiratory requirements (high flow, non-invasive ventilation, mechanical ventilation, or ECMO). The use of baricitinib may be an alternative for patients in whom steroids are not feasible. If there are concerns for a dysregulated immune/inflammatory response, then biologics, such as baricitinib or tocilizumab, can be considered for children older than 2 years of age (77).

Previously, several monoclonal antibodies have been used for outpatient treatment of mild SARS-CoV-2 infections in children ≥12 years and older, but with the currently circulating variants, there is no available monoclonal antibody for neither treatment nor pre-exposure prophylaxis.

### Prevention strategies

#### Influenza

In a 2014 guideline, the Infectious Diseases Society of America recommends the inactivated influenza vaccine (IIV) for children older than 6 months of age with hematologic and solid organ malignancies, except in patients receiving anti B-cell antibodies or intensive chemotherapy due to low vaccine response in these populations (104). However, HCT candidates and those receiving intensive chemotherapy are at increased risk for complications related to influenza; therefore, given the seasonality of influenza and the low side effect profile of IIV in cancer patients, IIV may be considered to provide some adaptive immunity against seasonal influenza. Given the low response to the vaccine, some institutions require 2 doses at least 28 days apart, in this population (105). Vaccination is recommended for all family members, close contacts, and healthcare workers who care for immunocompromised children.

Chemoprophylaxis with NAIs is recommended for any child at high risk for influenza complications after close contact with an influenza positive patient or during an outbreak, regardless of vaccination status (76). Chemoprophylaxis with NAIs should be started within 48 h of exposure for 7 day duration (76). However, given the risk for poor outcomes in this population, prophylaxis should be considered even if beyond 48 h from exposure.

#### Respiratory syncytial virus

Palivizumab is a humanized monoclonal antibody against the RSV fusion (F) glycoprotein, which has been in the market since 1998 for high-risk infants and young children. However, it requires costly, monthly injection during RSV season. The discovery of RSV pre-fusion F structure unveiled an epitope site targeted by neutralization antibodies and has been the target of current RSV vaccines and alternative therapies.

Nirsevimab is a novel monoclonal antibody that targets the RSV pre-fusion glycoprotein and lasts 6 months after one intramuscular injection. Studies in preterm and immunocompetent infants showed lower rates of RSV LRTI in the nirsevimab group compared to placebo (106). As of July 2023, the FDA has approved nirsevimab in infants born during or entering their first RSV season and in children up to 24 months of age who remain vulnerable to severe RSV disease through their second RSV season, including immunocompromised children undergoing HCT (107). The MUSIC study evaluated the safety and tolerability for nirsevimab in immunocompromised children. It was an open label phase 2b clinical trial involving 100 participants. Serious adverse events were uncommon and no medically attended RSV-LRTI was observed during the study period (108).

Currently, there are two RSV vaccines available for adults greater than 60 years of age, one of which is also available for pregnant people. However, none of these are available for children. Further studies are needed to evaluate the efficacy of RSV vaccines in pediatric HCT candidates and recipients.

#### SARS-CoV-2

There are three approved FDA SARS-CoV-2 vaccines in children. The mRNA vaccines from Pfizer/BioNTech and Moderna are approved for children 6 months of age or older, and the protein subunit vaccine from Novavax is approved for children ≥12 years of age. The efficacy of these vaccines has varied in immunocompetent children (109, 110). There is limited data on vaccine immunogenicity in immunocompromised and pre-HCT children, but in a study of immunocompromised adults, there were lower rates of seroconversion in HCT and solid organ recipients (111). Despite the lower vaccine efficacy in immunocompromised patients, vaccination of all cancer patients and HCT candidates and recipients older than 6 months of age as well as all family members and close contacts should be considered. Updated boosters targeting the Omicron XBB.1.5 subvariant have been developed, authorized, and recommended. For the latest up to date guidance on updated vaccines, please refer to the CDC recommendations (110). There are no pre-exposure prophylaxis agents for SARS-CoV-2 approved in the US at the time of this publication.

### Summary: Although limited options are available, preventative interventions and treatments should be considered preHCT to mitigate the burden and potential adverse outcomes related to these viruses

## Future directions

The COVID-19 pandemic has provided valuable lessons for the transplant infectious diseases community. Identifying patients at risk for progression to severe disease from a respiratory virus and providing appropriate care can prevent hospitalization and decrease mortality. The rapid implementation of the SARS-CoV-2 vaccine and the development of therapies (monoclonal antibodies and antivirals) have likely saved many immunocompromised children's lives. However, the same advancements are needed for other respiratory viruses and identifying risk factors as well as biomarkers for progression to severe disease is necessary. Additionally, further multicenter studies and randomized control trials that include immunocompromised children are needed to allow for more equitable, data-driven recommendations in this vulnerable population, especially in regard to pre-HCT RVIs.
